# The Na/K-ATPase α1 and c-Src form signaling complex under native condition: A crosslinking approach

**DOI:** 10.1038/s41598-020-61920-4

**Published:** 2020-04-07

**Authors:** Ying Nie, Fang Bai, Muhammad A. Chaudhry, Rebecca Pratt, Joseph I. Shapiro, Jiang Liu

**Affiliations:** 1 Department of Biomedical Sciences, Joan C. Edwards School of Medicine, Marshall University, Huntington, WV 25755 USA; 2 Department of Medicine, Joan C. Edwards School of Medicine, Marshall University, Huntington, WV 25755 USA

**Keywords:** Membrane proteins, Ion channel signalling

## Abstract

The protein-protein interactions amongst the Na/K-ATPase α1 subunit, c-Src, and caveolin-1 (cav-1) are essential for the Na/K-ATPase signaling functions. However, there are arguments concerning the interaction model. The present study aims to clarify the interactions amongst the endogenous native proteins in live cells under native resting condition. Under native condition, Blue Native-PAGE and Blue Native-PAGE/SDS-PAGE 2D analyses demonstrated co-existence of the α1 subunit and c-Src in same protein complex, as well as a direct interaction between the α1 subunit and c-Src. By comparison of cleavable and non-cleavable cysteine-cysteine crosslinked samples, capillary immunoblotting analysis demonstrated that depletion of Src kinase family members (c-Src, Yes, and Fyn) or cav-1 clearly reduced the interactions of the α1 subunit with proteins, but depletion of cav-1 did not affect the interaction of c-Src with the α1 subunit. The data indicated that there are direct interactions between the α1 subunit and c-Src as well as between the α1 subunit and cav-1, but argued about the interaction between c-Src and cav-1 under the condition. Furthermore, the data also indicated the existence of different protein complexes containing the α1 subunit and c-Src, which might have different signaling functions.

## Introduction

The P-type ATP-hydrolyzing enzyme Na/K-ATPase (EC 3.6.3.9) is an integral membrane protein that was first discovered by Jens C. Skou^1^. The Na/K-ATPase was first recognized as the primary ion transporter to maintain the electrochemical sodium gradient across cell membrane by using an ATP/ADP-dependent phosphorylation/dephosphorylation process that causes conformational changes in two states of the enzyme, E1(P) and E2(P). In the last two decades, the Na/K-ATPase was also recognized as a receptor, signal transducer, and scaffolding protein through multiple protein-protein interactions^2–10^. Binding of ouabain (one of the cardiotonic steroids) to the Na/K-ATPase α1 subunit or increasing of reactive oxygen species (ROS) initiate signaling pathways. This leads to increases in oxidative modification of the α1 subunit and intracellular calcium concentration, and other effects. Receptors, signaling molecules, cytosolic proteins, and membrane structural proteins can interact with the α1 subunit through multiple structural binding motifs found in the α1 subunit^10,11^. These include, but are not limited to, c-Src, epidermal growth factor receptor (EGFR), phospholipase C (PLC), phosphoinositide 3-kinases (PI3K), inositol trisphosphate receptor (IP3Rs), ankyrin, adducin, and caveolin-1 (cav-1). The Na/K-ATPase signaling pathways have been demonstrated in different type of cells and animal models^12–15^.

In recent years, there are different proposed “working” models which explain the mechanisms underlying the activation of the Na/K-ATPase signaling function. The first model is the direct interaction of the α1 subunit with c-Src, which forms a functional Na/K-ATPase/c-Src signaling receptor complex in caveolae^8,16^. In this model (Model-1, proposed by Dr. Xie’s group), the α1 subunit provides the ligand binding sites, the α1-associated c-Src provides the kinase moiety, and the cav-1 functions as an anchor to enrich the signaling partners in caveolae. A second model (Model-2, proposed by Dr. Karlish’s group) proposes that c-Src only transiently interacts with a protein complex formed between the α1 subunit and cav-1^17^. A third model is that c-Src activation is primarily a consequence of an ATP-sparing effect, depending on ATP/ADP ratio, without interaction between the α1 subunit and c-Src^18,19^ (proposed by Dr. Koenderink’s group). This third model will not be discussed since the protein-protein interaction is the focus in present study. However, a common charateristic in these models is that the E2(P) conformational state of the Na/K-ATPase is favored and stablized by Na/K-ATPase inhibitors (ouabain, vanadate, oligomycin etc.) and energy status (ATP/ADP ratio). Though the dynamic conformational changes can affect the formation of the signaling complex, it is quite clear that c-Src activation is one of the most proximal steps in the Na/K-ATPase signaling since the Na/K-ATPase itself lacks tyrosine kinase activity. The discrepancies between Model-1 and Model-2 could be attributed to the diferent experimental designs and conditions, since the interaction of the α1 subunit with c-Src or cav-1 might also require other protein(s) that are not present in some experimental conditions as suggested in^17^.

The Na/K-ATPase has emerged as a therapeutic target for different pathological states, largely based on its signaling axis [reviewed in^2,20–27^]. Even though there are plausible reasoning for each “working” model, it is imperative to better understand the underlying mechanisms in live cells under native resting condition.

## Results

### Depletion of cav-1, but not Src kinase family, increased ion-transport activity of the Na/K-ATPase

**Figure 1:**
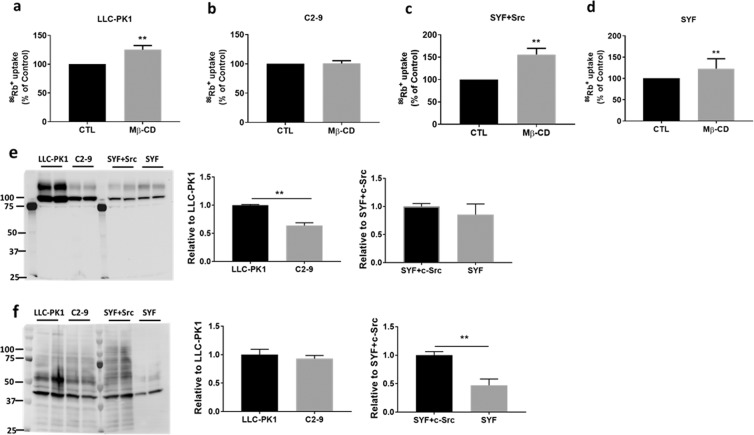
The effect of Mβ-CD on ouabain-sensitive Na/K-ATPase ion-transport activity (^86^Rb^+^ uptake) and characteristics in different cell types: Before initiation of ^86^Rb^+^ uptake, cells were pretreated with 10 mM of Mβ-CD for 30 min at 37 °C. The ^86^Rb^+^ uptake was calibrated with protein content. Data were expressed as the percentage of control ouabain-sensitive ^86^Rb^+^ uptake (Mean ± SD). n = 8–12 independent experiments (each performed in triplicate) for each cell type. **(a)** LLC-PK1, n = 9. **(b)** C2-9, n = 8. **(c)** SYF + c-Src, n = 12. **(d)** SYF, n = 8. **p < 0.001 *vs*. control (CTL). Comparison of cell surface Na/K-ATPase α1 subunit by cell surface biotinylation (**e**) and protein carbonylation (**f**) between LLC-PK1 and C2-9 cells, and between SYF and SYF + c-Src cells. Data were presented as Mean±SD, n = 3. **p < 0.05 *vs*. LLC-PK1 or SYF + c-Src cells.

It has been shown that depletion of either cholesterol by methyl β-cyclodextrin (Mβ-CD) or cav-1 moves the α1 subunit and c-Src out of caveolae, which leads to significantly reduced interactions amongst the α1 subunit, c-Src, and cav-1, as well as ouabain-stimulated Na/K-ATPase signaling^16^. For the Model-1, the bindings of c-Src SH2 and KD domains to the α1 CD2 and ND1 segments to form a signaling complex have been demonstrated^8^. However, there are very interesting questions arguing the accessibility and fitting of c-Src, as a whole 60 kDa molecule, into the α1 subunit under native condition^18,28^. Even though there is no clear answer, a functional analysis of ouabain-sensitive ^86^Rb^+^ uptake should shed light on the possibilities. To do so, cells were pretreated with agent’s vehicle (as control), or with 10 mM Mβ-CD for 30 min at 37 °C. As shown in Fig. 1, Mβ-CD treatment increased ouabain-sensitive ^86^Rb^+^ uptake in LLC-PK1 but not in cav-1-knockdown C2-9 cells, which supports the observation that cav-1 depletion can move the α1 subunit and c-Src from the caveolae-resided “signaling” pool to an “ion-pumping” pool, leading to increase in ion-transport activity and reduction of signaling^16,29^. However, Mβ-CD treatment increased ^86^Rb^+^ uptake in both SYF + c-Src and SYF cells. The data suggested that Mβ-CD-mediated increase in ion-transport activity is in a cav-1-dependent, but c-Src-independent manner.

In comparison with LLC-PK1 cells, C2-9 cells showed lower abundance of the Na/K-ATPase α1 subunit on cell surface (~36% reduction), with similar protein carbonylation level and cellular ATP level (29.09 ± 1.56 nM ATP/mg protein, vs. 28.30 ± 1.73 nM ATP/mg protein in LLC-PK1 cells, n = 6). Since the “signaling” pool was still present when the α1 subunit was reduced to ~50%^16,29^, it is reasonable to assume that it is depletion of cav-1, but not reduction of α1 subunit alone, will affect the signaling function in C2-9 cells. This is further supported by the observation that P-11 cells (expressing mock vector) had similar cell surface α1 subunit as seen in C2-9 cells (expressing cav-1 siRNA vector), and ouabain can stimulate the signaling and endocytosis of the α1 subunit in P-11 cells but not in C2-9 cells^30^. SYF and SYF + c-Src cells were developed from mouse embryo fibroblasts. Both cell lines are viable but SYF cells showed reduced mobility and deficiencies in signaling and embryogenesis^31^. In comparison with SYF + c-Src cells, SYF cells showed similar abundance of cell surface α1 subunit, with significant lower protein carbonylation level and cellular ATP level (15.05 ± 1.66 vs. 32.180 ± 2.80 nM ATP/mg protein in SYF + c-Src cells). This suggests that c-Src is crucial in ouabain-stimulated Na/K-ATPase signaling since ouabain can activate the signaling in SYF + c-Src cells but not in SYF cells^16^.

### Validation of crosslinking procedure and capillary immunoblotting analysis with crosslinked samples

**Figure 2:**
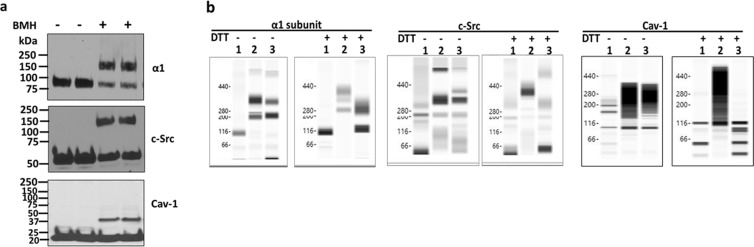
Validation of crosslinking and capillary immunoblotting analysis of LLC-PK1 cells: (**a**) LLC-PK1 cells were processed with or without BMH crosslinking as described in the Materials and Methods. Whole cell lysates were prepared with Nonidet P-40 lysis buffer, and then processed for traditional immunoblotting analysis under denature condition (heated at 60 °C for 30 min for the α1 subunit, and 95 °C for 5 min for c-Src and cav-1 detection) with SDS-PAGE. 60 µg of total protein per sample was used. After transferring, PVDF membranes were cut below the molecular weight of monomers, i.e., below 75 kDa for the α1 subunit, below 50 kDa for total c-Src, and below 20 kDa for cav-1. ECL was used to develop blots. n = 2–3. **(b)** After crosslinking, whole cell lysates of LLC-PK1 cells were prepared with 1x Native-PAGE sample buffer (with 2% DDM). The lysates (3 μg protein/sample) were treated without or with DTT (final concentration 40 mM) plus SDS (final concentration 1%), and then processed side-by-side on the same 66–440 kDa separation module and immunoblotted with detection module (Wes system, ProteinSimple) for capillary immunoblotting analysis against the α1 subunit, total c-Src, and cav-1. For DTT/SDS treatment, samples were heated at 60 °C for 30 min (for the α1 subunit) or 95 °C for 5 min (for c-Src and cav-1). Lane 1, control with mocking crosslinking process; Lane 2, BMH-crosslinked; and Lane 3, DTME-crosslinked. n = 5.

In the initial studies, we use traditional SDS-PAGE to test the efficiency of crosslinker bismaleimidohexane (BMH). Both BMH (spacer arm 13.0 Å) and dithiobismaleimidoethane (DTME) (spacer arm 13.3 Å) are homobifunctional, maleimide crosslinker for conjugation between sulfhydryl groups (-SH) in cysteine residues. While BMH is non-cleavable, DTME contains a disulfide (-S-S-) bond in the middle of its space arm that makes it cleavable with reducing agents such as dithiothreitol (DTT). To test the crosslinking procedure, Porcine renal proximal tubule LLC-PK1 cells were crosslinked with or without BMH. Whole cell lysates were prepared with the Nonidet P-40 lysis buffer (containing 1% Nonidet P-40, 0.25% sodium deoxycholate, 50 mM NaCl, 50 mM HEPES, 10% Glycerol, pH 7.4, and Halt Protease and Phosphatase Inhibitor Cocktail), and then denatured with sample buffer (Bio-Rad, with 100 mM DTT) and applied to 10% SDS-PAGE immunoblotting analysis. In contrast to control samples which showed a single band when immunoblotted for the α1 subunit (~100 kDa), total c-Src (~60 kDa), and cav-1 (~20 kDa), BMH-crosslinked samples showed two bands, in which one was in the same position as seen in control samples, and an additional band appearing at a higher molecular mass, i.e., ~150 kDa for the α1 subunit and c-Src as well as ~40 kDa for cav-1 (Fig. 2a). These indicated that the crosslinking procedure worked as expected, but only part of the target proteins was crosslinked. Since DTME-crosslinked samples will be cleaved in the presence of DTT, these samples were not included in this validation experiment.

To explore the possible protein-protein interactions amongst the α1 subunit, c-Src, and cav-1 in live LLC-PK1 cells, whole cell lysates were prepared with NativePAGE sample buffer for capillary immunoblotting analysis (Wes system, ProteinSimple). Control and crosslinked samples were processed with or without DTT/SDS (40 mM and 1%, final concentration, respectively), heated at 60 °C for 30 min for the α1 subunit detection or 95 °C for 5 min for c-Src and cav-1 detection. Figure 2b (left panel) shows the α1 immunoblotting analysis. Without DTT/SDS treatment, there were multiple bands around 100 kDa (control), 200 kDa, and 350 kDa (molecular weight data was extracted from Compass for SW, ProteinSimple) in both BMH- and DTME-crosslinked samples. When the same samples were treated with DTT/SDS, non-cleavable BMH-crosslinked sample showed a similar pattern as seen in samples without DTT/SDS treatment. On the other hand, in cleavable DTME-crosslinked sample treated with DTT/SDS, the signal density of the 350 kDa band was significantly decreased, accompanying a significant increase in signal densities of 100 kDa and 200 kDa bands. When the same samples were applied for total c-Src (Fig. 2b, middle panel)) and cav-1 (Fig. 2b, right panel) analysis, similar redistribution of immunoblotting patterns were observed as seen in the α1 subunit. The data suggested that both BMH and DTME were efficient to crosslink proteins into multiple protein-protein complexes and could be used as a tool for comparison. It is worth noting that the migrations of DTT/SDS-treated samples were slower than the non-DTT/SDS-treated samples. This suggested that DTT/SDS treatment might reduce the disulfide bonds which can cause conformation changes^32^. This phenomenon can also be attributed to the changes of protein hydrophobicity^33^ and detergent binding^34^.

### The α1 subunit and c-Src form protein-protein complex under native condition with and without crosslinking

**Figure 3:**
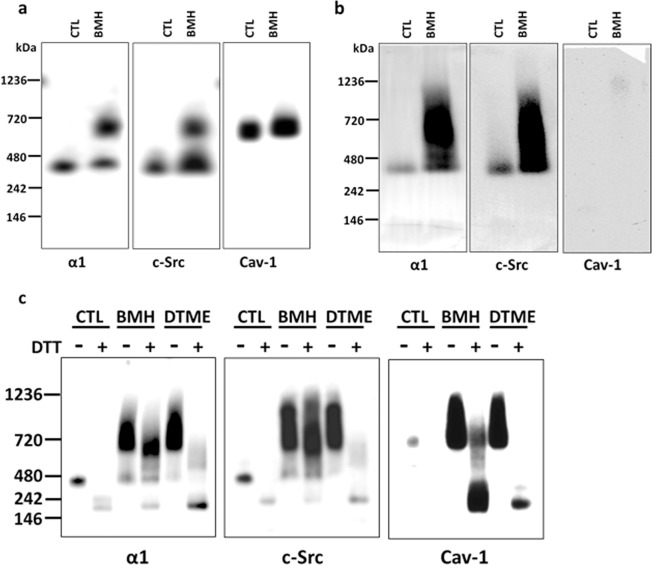
BN-PAGE analysis of LLC-PK1 and C2-9 cells: The α1 subunit and c-Src form protein-protein complex under native condition that is not dependent on cav-1. Crosslinking and preparation of whole cell lysate with Native-PAGE sample buffer were performed as described for BN-PAGE in the Materials and Methods. NativeMark unstained protein standard was located by Ponceau S staining after transferring to PVDF membrane. Control (CTL, with mocking crosslinking process without crosslinkers) and BMH- or DTME-crosslinked samples (25 μg protein/sample) were processed side-by-side and immunoblotted for the α1 subunit, c-Src, and cav-1, respectively. For **(a**,**b)**, the same CTL and crosslinked samples were separated into 3 groups (each group contains one CTL and crosslinked samples and separated by NativeMark protein standard between groups) and run in the same gels. After transferring, the PVDF membrane was cut into the 3 groups and immunoblotted against each antibody individually. **(a)** BN-PAGE analysis of control (CTL) and BMH-crosslinked samples of LLC-PK1 cells**. (b)** BN-PAGE analysis of control (CTL) and BMH-crosslinked samples cav-1 depleted C2-9 cells. **(c)** Each sample (CTL, BMH- and DTME-crosslinked sample) of LLC-PK1 cells was treated with or without DTT/SDS (100 mM DTT with 1% SDS, final concentration, respectively). For DTT/SDS treatment, samples were heated at 60 °C for 30 min (for the α1 subunit) or 95 °C for 5 min (for c-Src and cav-1). FluorChem M imager system (ProteinSimple) was used to detect blot signals. n = 3–4.

Blue Native PAGE (BN-PAGE) analysis is a well-established method to investigate membrane-associated multiple protein complexes under native condition^35–37^. To eliminate the possible variation between gel running, control and crosslinked samples were run side-by-side in the same BN-PAGE gel. After transferring of proteins, the PVDF membranes were fixed with 8% acetic acid, and then cut into membrane strips to immunoblot for the α1 subunit, c-Src, and cav-1. As shown in Fig. 3a (for original blots of Fig. 3, please see Supplementary Information, Fig. S1), the control and BMH-crosslinked samples showed immuno-reactivity with the α1 subunit and c-Src just below 480 kDa marker (named as the 480-band since estimation of the exact molecular mass is difficult). In addition, the BMH-crosslinked sample clearly showed an additional and much stronger signal band just below 720 kDa marker (named as the 720-band) when immunoblotted for α1 or c-Src. Interestingly, immunoblotting for cav-1 showed predominantly in the 720-band in control and BMH-crosslinked samples. However, there was no obvious immuno-reactivity in the 480-band even after extensive exposure. This pattern was also observed in control and DTME-crosslinked samples (data not shown). The presence of cav-1 in high molecular homo-oligomers by self-association^38,39^ has been demonstrated in COS-7 cells^40^ and ACHN cells^41^, in whole cell lysates solubilized with n-dodecyl-β-D-maltoside (DDM). The data indicated that crosslinking with both BMH and DTME was sufficient to form a new protein complex at the 720-band that contains the α1 subunit and c-Src.

The data also argued about the involvement of cav-1 regarding the formed complex in the 480-band. The predominant cav-1 immuno-reactivity in the 720-band in both control and crosslinked samples suggested that most of cav-1 did not form a complex in the 480-band with the α1 subunit/c-Src in native control condition. The predicted molecular weight should be much higher than 720 kDa if the α1 subunit/c-Src complex (the 480-band) crosslinked with cav-1 self-associated homo-oligomers (the 720-band). The absence of cav-1 in the α1 subunit/c-Src complex (the 480-band) was further supported by BN-PAGE analysis of cav-1 knockdown C2-9 cells (Fig. 3b), in which the α1 subunit and c-Src showed a similar immuno-reactivity pattern in control and BMH-crosslinked samples as seen in LLC-PK1 cells. However, it could not exclude the possibility that the crosslinked complex(s) is beyond the BN-PAGE separation range.

Both the 720-band and 480-band were processed for mass spectrometry analysis, which was searched against a pig database for Na/K-ATPase, c-Src, and cav-1 (Table 1). While the Na/K-ATPase α1 and β1 subunits were present in both the 720-band and 480-band in both control and BMH-crosslinked samples, cav-1 was present in the 720-band but not detectable in the 480-band. Interestingly, while c-Src was detected in the 480-band but not the 720-band in control samples, c-Src was present in both the 720-band and 480-band in BMH-crosslinked samples. The data suggests that the α1 subunit and c-Src are capable to form a complex without cav-1 under native control condition (the 480-band), and BMH-crosslinking might form a new complex containing the α1 subunit, c-Src, and cav-1 (the 720-band).

**Table 1:** Mass spectrometry analysis of the 720 kDa and kDa bands of control and BMH-crosslinked samples.

Accession Number	Alternate ID	CTL-720kD	CTL-480kD	BMH-720kD	BMH-480kD
		Protein Identification Probability			
D2WKD6_PIG (+ 2)	ATP1A1	100%	100%	100%	100%
AT1B1_PIG	ATP1B1	100%	100%	100%	100%
A0A286ZJQ9_PIG (+1)	CAV1	100%	0	97%	0
F1SEK8_PIG (+2)	SRC	0	97%	100%	97%

### Cav-1 is not likely crosslinked with the α1/c-Src complex

To further explore the protein complexes at the 480-band and the 720-band, control, BMH- and DTME-crosslinked samples were divided into two equal aliquots. One aliquot was without any treatment, and the other aliquot was treated with DTT/SDS (100 mM/1%, final concentration, respectively) and heated at 60 °C for 30 min (for the α1 detection) or at 95 °C for 5 min (for c-Src and cav-1 detection). As shown in Fig. 3c, compared to non-DTT/SDS treated samples, DTT/SDS treatment disrupted the α1 subunit/c-Src protein complexes in control and DTME-crosslinked samples, but not in BMH-crosslinked samples. Interestingly, in both BMH- and DTME-crosslinked samples treated with DTT/SDS, the disappearance of cav-1 signal at the 720-band strongly indicated that cav-1 was largely not affected by Cys-Cys crosslinking. This also suggested that even though there might be a protein-protein interaction between cav-1 and the α1 subunit or c-Src, the interaction(s) are not likely through a Cys-Cys interaction since the interaction(s) are sensitive to DTT/SDS treatment. This observation is supported with the reports that cav-1 homo-oligomers were sensitive to boiling heat in the presence of SDS and β-mercaptoethanol^38,39^.

### The α1 subunit and c-Src form protein-protein complex under native condition: Two-dimensional (2D) analysis

**Figure 4:**
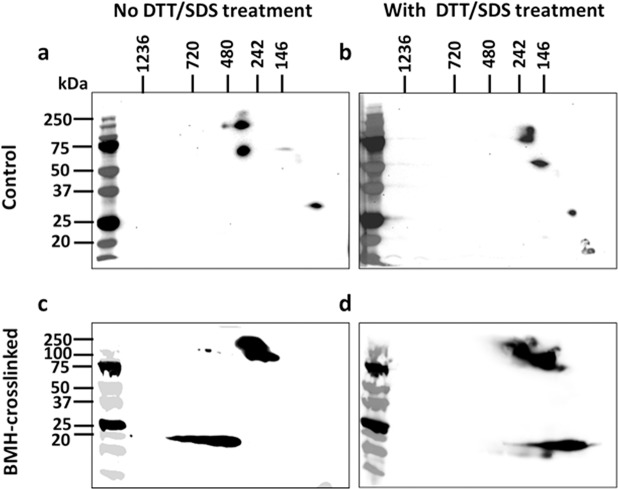
BN-PAGE/SDS-PAGE 2D gel electrophoresis analysis – the α1 subunit and c-Src form a protein complex: Preparation of whole cell lysate with Native-PAGE sample buffer were performed as described for BN-PAGE in the Materials and Methods. The experiments were performed as described for BN-PAGE/SDS-PAGE 2D Gel Electrophoresis in the Materials and Methods. Control and BMH-crosslinked samples of LLC-PK1 cells (50 μg/sample) was treated with or without DTT/SDS (100 mM DTT with 1% SDS, final concentration, respectively). For DTT/SDS treatment, samples were heated at 60 °C for 30 min (for the α1 subunit) or 95 °C for 5 min (for c-Src and cav-1). **(a)** Control sample without DTT/SDS pretreatment. **(b)** Control sample with DTT/SDS pretreatment. **(c)** BMH-crosslinked sample without DTT/SDS pretreatment. **(d)** BMH-crosslinked sample with DTT/SDS pretreatment. Please note the vertical alignment of the α1 subunit and c-Src in **(a)** but not in **(b)**, which indicated that both proteins were from the same protein complex. Comparing to control **(a**,**b)**, disappearance of c-Src in **(c)** and **(d)** indicated the crosslinking between the α1 subunit and c-Src. n = 3–4.

Since proteins shown at the same molecular weight position could not confirm the interaction by one dimensional Western blotting analysis, the BN-PAGE (as first dimension) and SDS-PAGE (as second dimension) 2D analysis was employed to further identify the α1 subunit, c-Src, and cav-1 in the protein complexes shown in the 480-band and the BMH-crosslinked 720-band. As shown in Fig. 4a (for original blots of Fig. 4, please see Supplementary Information, Fig. S2), in non-DTT/SDS-pretreated control sample in which contains protein complex, both the α1 subunit and c-Src blotting spots, but not cav-1, were vertically aligned. In contrast, the DTT/SDS-pretreated sample which contained protein monomers, α1, c-Src, and cav-1 signals were moved to the right side (Fig. 4b). According to the principals of BN-PAGE/SDS-PAGE 2D analysis^37,42^, this indicated that both the α1 and c-Src, but not cav-1, were in the same protein complex under the conditions. In the same 2D analysis with BMH-crosslinked sample (Fig. 4c,d), the c-Src blotting spot disappeared along with the presence of a strong blotting spot around 100–250 kDa (presented as crosslinked α1 and c-Src) that was not sensitive to DTT/SDS pretreatment. However, the cav-1 blotting spot was still not vertically aligned with the crosslinked α1/c-Src blotting spot (Fig. 4c) and moved to right with DTT/SDS pretreatment (Fig. 4d). Again, it is worth noting that the samples, pretreated with or without DTT/SDS, also exhibited different migration rate as shown in both Figs. 2b and 3c.

### Capillary immunoblotting analysis of protein-protein interactions

With obvious advantages, a limitation of BN-PAGE is the widespread chemiluminescent signals in immunoblotting analysis which could be from one or multiple complexes. Moreover, in BN-PAGE, it was widely recognized that different conformation with different protein partners affected native folding and antigen presentation of specific proteins, and thus antigen-specific antibody binding and immunoblotting signal. This was seen between control and crosslinked samples (Figs. 3 and 4), showing much stronger chemiluminescent signals in crosslinked samples. Based on the results from the BN-PAGE, a capillary immunoblotting analysis, a method gives more separation detail, was employed to investigate the role of cav-1 and c-Src in the formation of protein complexes.

**Figure 5:**
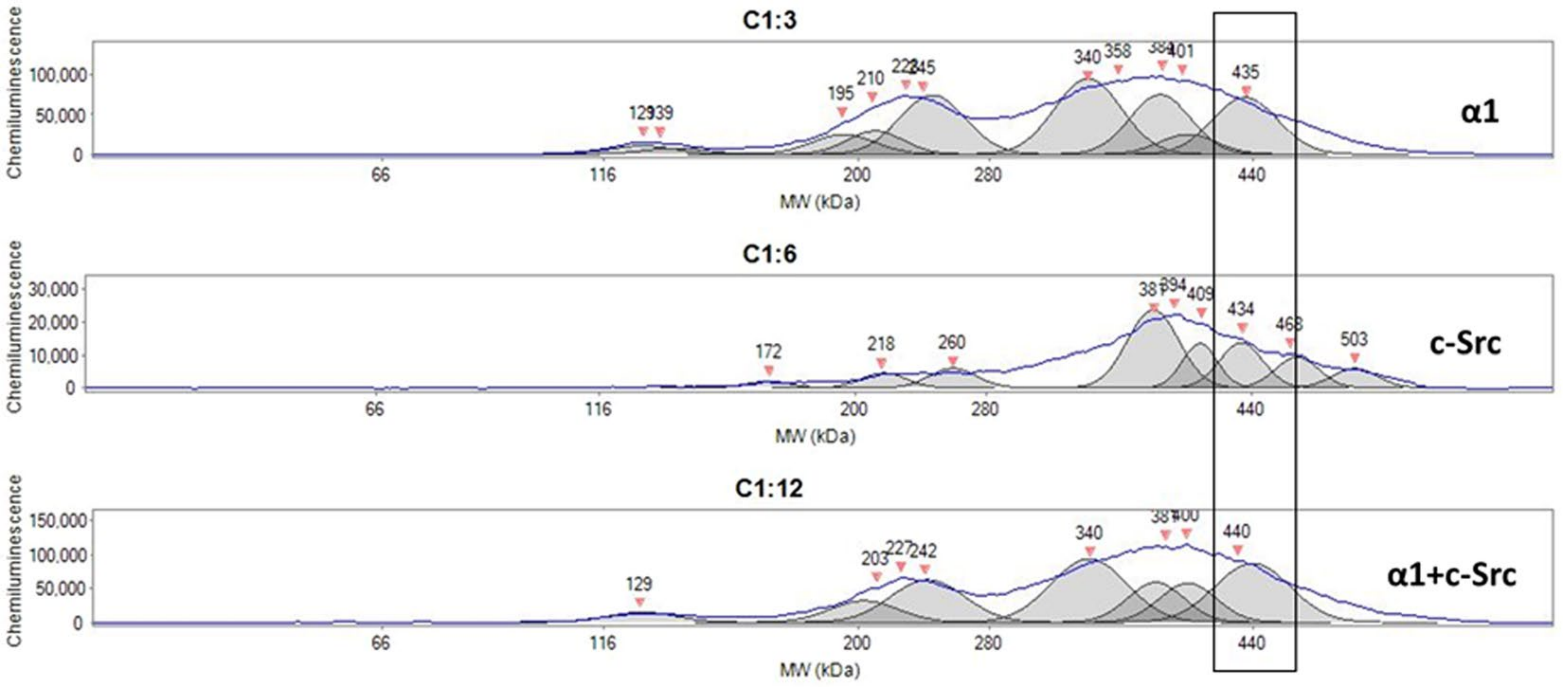
Immunoblotting analysis for the α1 subunit, c-Src, and α1 + c-Src in LLC-PK1 cells by capillary immunoblotting: Preparation of whole cell lysate with Native-PAGE sample buffer were performed as described for BN-PAGE in the Materials and Methods. Same DTT/SDS–treated, BMH-crosslinked samples (3 μg protein/sample) of LLC-PK1 cells were electrophoresed side-by-side on the same 66–440 kDa separation module and immunoblotted with detection module (Wes system, ProteinSimple) with antibodies against the α1 (upper panel), c-Src (middle panel), and α1 + c-Src (lower panel, mixture of the two antibodies with same dilutions for α1 alone and c-Src alone) separately. For DTT/SDS treatment, samples were heated at 60 °C for 30 min (for the α1 subunit and α1 + c-Src) or 95 °C for 5 min (for c-Src). Please note there are multiple peaks. n = 3.

To test the feasibility of the method, BMH-crosslinked samples (3 µg/sample) of LLC-PK1 cells were treated with DTT/SDS and processed side-by-side for capillary immunoblotting spectrum analysis, using antibodies against the α1, c-Src, and a mixture of α1 and c-Src (same antibody concentrations as in α1 alone and c-Src alone). As shown in Fig. 5, a peak (~434–440 kDa) area immunoblotted with antibody mixture (lower panel) showed a chemiluminescent density that is close to the sum of antibody against the α1 alone (upper panel) and antibody against c-Src (middle panel). There were also two groups of peaks around 200–240 kDa and 340–400 kDa, which could explain the widespread chemiluminescent signals shown in BN-PAGE. Since BMH-crosslinked protein-protein interaction is not sensitive to DTT/SDS treatment, this also suggested that α1 and c-Src are able to form different protein-protein complexes that are of interest for future identification.

### Depletion of Src kinase family altered interaction pattern of the α1 subunit with other proteins

**Figure 6:**
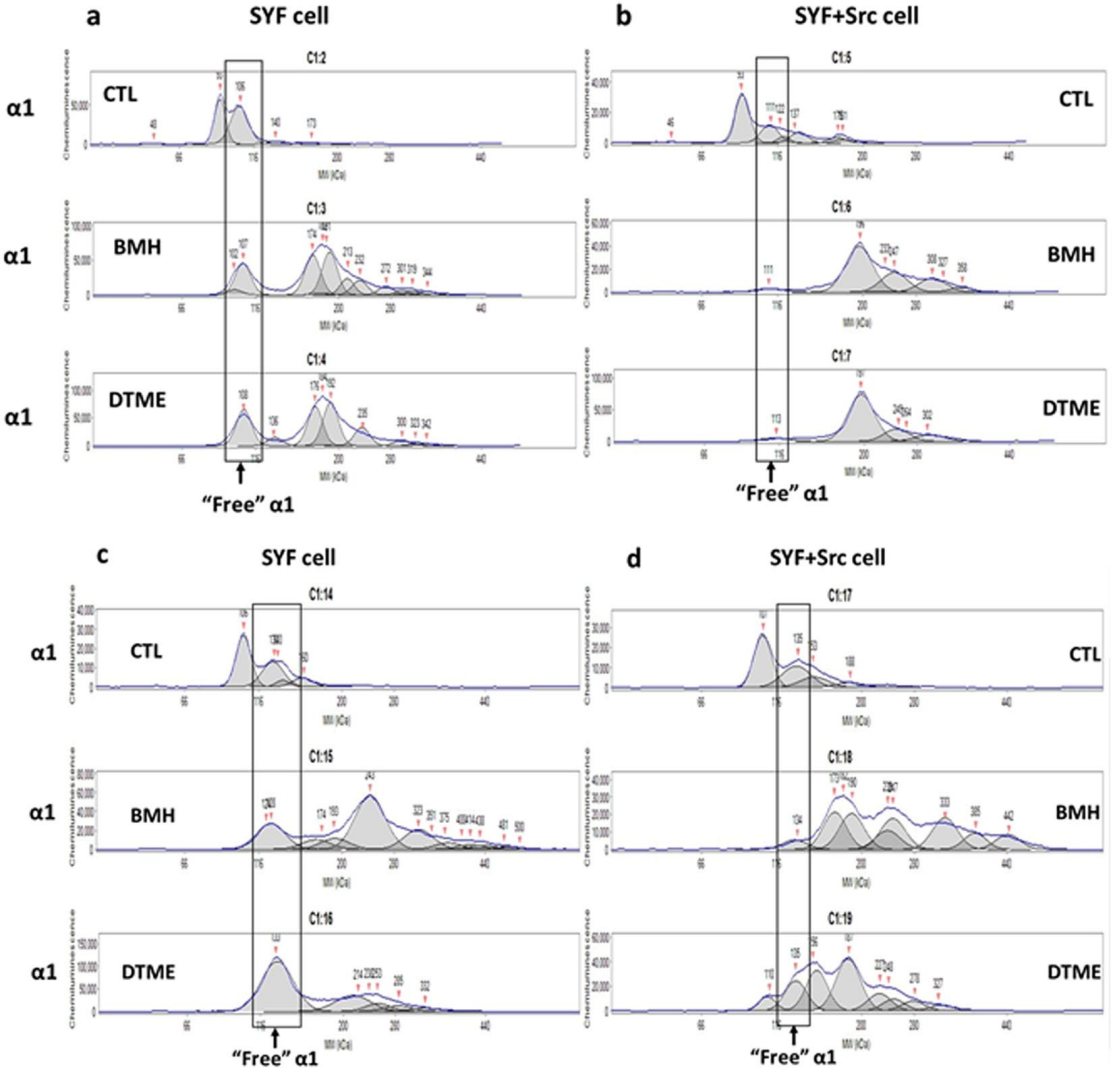
Knockout of Src kinase family reduces binding of the α1 subunit with other proteins - comparison of α1 subunit-c-Src binding pattern between SYF and SYF + c-Src cells by capillary immunoblotting: Preparation of whole cell lysate with Native-PAGE sample buffer were performed as described for BN-PAGE in the Materials and Methods. Samples (3 μg protein/sample) were electrophoresed side-by-side on the same 66–440 kDa separation module and immunoblotted with detection module. For DTT/SDS treatment, samples were heated at 60 °C for 30 min for the α1 subunit detection. Control (CTL), BMH- and DTME-crosslinked samples without DTT/SDS pretreatment of SYF cells **(a)** and SYF + c-Src cells **(b)**, or with DTT/SDS pretreatment of SYF cells **(c)** and SYF + c-Src cells **(d)**, and then immunoblotted for α1 subunit. Please note the differences of α1 pattern between SYF and SYF + c-Src cells. n = 3.

To explore the role of c-Src in the formation of the Na/K-ATPase signaling complex, triple Src kinase (c-Src, Yes, Fyn)-null SYF cells and c-Src rescued SYF cells, SYF + c-Src cells, were used to demonstrate the involvement of c-Src. Whole cell lysates (3 µg/sample) of SYF and SYF + c-Src cells were processed side-by-side to immunoblot for the α1 subunit. In non-DTT/SDS-treated samples, a large portion of “free” α1 monomer (~100 kDa) in SYF cells was not crosslinked by BMH or DTME, comparing to control sample (Fig. 6a). In contrast, in SYF + c-Src cells, most of the “free” α1 was crosslinked by BMH or DTME, demonstrated by the disappearance of the “free” α1 in BMH- and DTME-crosslinked samples (Fig. 6b). In DTT/SDS-treated samples, SYF samples showed obvious “free” α1 monomers in control and crosslinked samples (Fig. 6c), as seen in non-DTT/SDS-treated samples (Fig. 6a). However, in SYF + c-Src cells, DTT/SDS-treated BMH-crosslinked sample (Fig. 6d, middle panel) showed similar distribution pattern as seen in non-DTT/SDS-treated samples (Fig. 6b, middle panel), but DTT/SDS-treated, DTME-crosslinked sample showed disrupted complex formation, demonstrated by the reappearance of the “free” α1 monomers (Fig. 6d, lower panel) compared to non-DTT/SDS-treated samples (Fig. 6b, lower panel). The results indicated the important role of c-Src in the formation of the Na/K-ATPase signaling complex, i.e. the interaction between α1 and c-Src. Interestingly, in both BMH- and DTME-crosslinked samples with or without DTT/SDS treatment, there were other crosslinked complexes containing the α1 subunit in both SYF and SYF + c-Src cells, suggesting there were other proteins that were able to interact with the α1 subunit.

### Depletion of cav-1 altered interaction pattern of the α1 subunit, but not c-Src, with other proteins

**Figure 7:**
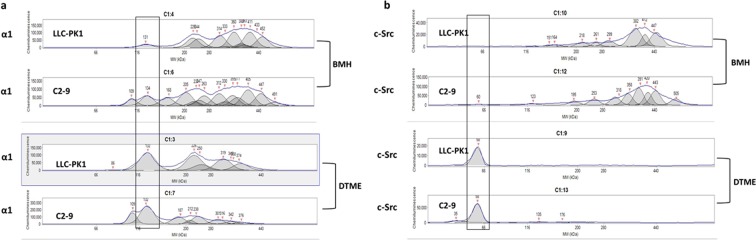
Comparison of α1 subunit-c-Src binding pattern between LLC-PK1 and C2-9 cells by capillary immunoblotting: BMH- and DTME-crosslinked samples (3 μg protein/sample) were pretreated with DTT/SDS, electrophoresed side-by-side on the same 66–440 kDa separation module, and immunoblotted against for the α1 subunit **(a)** or c-Src **(b)**. For DTT/SDS treatment, samples were heated at 60 °C for 30 min (for the α1 subunit) or 95 °C for 5 min (for c-Src). Please note the differences of α1 and c-Src patterns between LLC-PK1 and C2-9 cells. n = 4.

To test the role of cav-1, cav-1-knockdown C2-9 cells were compared with the parent wild-type LLC-PK1 cells. All samples were treated with DTT/SDS since DTT/SDS treatment disrupted the possible cav-1 interaction with α1/c-Src complex in both BMH- and DTME-crosslinked samples (Fig. 3c). Like SYF cell, there were a large portion of “free” α1 monomers that were not crosslinked by BMH in C2-9 cells (Fig. 7a, upper two panels) compared with BMH-crosslinked LLC-PK1 sample. However, DTT/SDS treatment did disrupt the protein complexes formed by DTME-crosslinking in both LLC-PK1 and C2-9 cells (Fig. 7a, lower two panels), indicating that cav-1 expression level affected the interaction of the α1 subunit with other proteins. Furthermore, these results show that the interaction between the α1 and cav-1 was not likely through a Cys-Cys crosslinking interaction. When same samples were immunoblotted for c-Src, the distribution of the immunoblotting patterns were very similar in both BMH-crosslinked sample (Fig. 7b, upper two panels) and DTME-crosslinked samples (Fig. 7b, lower two panels), indicating that cav-1 expression level did not affect the interaction of c-Src with other proteins and cav-1 might not be crosslinked with c-Src under the condition. Taken together, it indicated that cav-1 knockdown reduced the crosslinking of the α1 subunit with other proteins, but did not affect the crosslinking of c-Src with other proteins.

## Discussions

### The choice of crosslinkers

BMH and DTME are two cell membrane permeable crosslinkers that have reactive groups of maleimide on both ends, which can form a stable non-cleavable thioether bond between the maleimide groups and cysteine -SH groups. While the disulfide bond (–S-S-) placed in the middle of DTME’s space arm renders the crosslinked proteins cleavable by reducing agents such as DTT, absence of the –S-S- bond in BMH’s space arm makes it non-cleavable. This difference makes side-by-side comparison analysis possible. Another consideration of these two crosslinkers is the fact that they only crosslinks proteins through Cys-Cys interaction, which makes the crosslinking more specific.

In Model-1, the α1 subunit CD2 and ND1 segments binds to c‐Src SH2 domain and tyrosine kinase domain (KD)^4,8^. There are 23 Cys residues in the α1 subunit (UniProtKB# P05024_PIG), in which 2 Cys residues (Cys 209 and 247) were located in CD2 segment (aa 152–288) and 1 Cys (Cys426) was located in ND1 segment (aa 379–435). However, there is no Cys residue in the key residues of the proposed α1 caveolin-binding motif (CBM)^10^. c-Src (UniProtKB# K7GPR7_PIG) has 9 Cys residues, in which 3 Cys residues (Cys211, 264, and 271) were located in the SH2 domain (aa 174–271) and 6 Cys residues (Cys 303, 426, 509, 513, 522, and 524) were located in KD (aa 293–546). For cav-1 (UniProtKB# Q6RVA9_PIG), there are 3 Cys residues (Cys 133, 143, and 156) which are located outside the caveolin scaffolding domain (aa 61–101)^43^ that involves self-association to form high molecular homo-oligomers^39^.

### Interaction of the α1 subunit and c-Src in Na/K-ATPase signaling

The binding status of the α1 and c-Src is the centerpiece of the two proposed Na/K-ATPase signaling “working” models. The Model-1 showed the direct interaction of the α1 subunit with c-Src kinase which forms a functional Na/K-ATPase/c-Src signaling receptor complex^8^; and the Model-2 showed that c-Src transiently interacts with a stable protein complex formed between the α1 subunit and cav-1^17^. In both models, it is agreed that cav-1, by binding to the α1 subunit, functions as an anchoring protein to concentrate the “signaling” α1 subunit and its signaling partners in caveolae structure. Another common charateristic of these two models is that c-Src activation is a proximal step in the Na/K-ATPase signaling that was initiated by interaction, either stable or transient, between the α1 subunit and c-Src.

Different experimental approaches were performed to demonstrate these two models. The Model-1 was tested based on both cell-free system and live cells with different manipulation of the partner proteins (the α1 subunit, c-Src, and cav-1). On the other hand, the Model-2 was tested in a cell-free system, with over-expressed and purified partner proteins (including recombinant human Na/K-ATPase α1β1FXYD1 or porcine α1D369Nβ1FXYD1, human Src kinase, and human cav-1) as well as isolated plasmalemma microsomes, caveolae, and right-side out vesicles from rabbit kidney outer medulla^17^.

By applying BN-PAGE with whole cell lysate in native condition, control (with mocking crosslinking process) samples showed clear bands (the 480-band) when immunoblotted for the α1 subunit and c-Src (Fig. 3b). Both BMH- and DTME-crosslinked samples demonstrated an additional band (the 720-band) also showing the α1 subunit and c-Src (Fig. 3b). Interestingly, when immunoblotted against cav-1, control and crosslinked samples showed very similar blotting bands (the 720-band) in the same position as seen in crosslinked samples against the α1 subunit and c-Src, but not in the 480-band (Fig. 3b). Moreover, in 2D analysis, while control sample clearly showed that the α1 subunit and c-Src were from same protein complex (Fig. 4a,b), BMH-crosslinked sample showed a crosslinked α1/c-Src blotting spot with disapperance of c-Src blotting spot (Fig. 4c,d). The data demonstrated that the α1 subunit and c-Src formed a complex in the 480-band without croslinking, and a newly formed complex in the 720-band through Cys-Cys crosslinking.

In Model-1, the α1 subunit binds to c-Src through two pairs of domain biding (α1 CD2 segment with c-Src SH2 domain, and α1 ND1 segment with c-Src KD domain) under resting condition, and ouabain stimulated the release of c-Src KD from α1 ND1 segment that led to c-Src KD Tyr-418 phosphorylation (activation). In the Model-2 experiments, the Tyr-418 in purified c-Src was already phosphorylated in the isolation/purification process before binding experiments^17^, likely leaving only one pair of possible domain binding (i.e. α1 CD2 with c-Src SH2) under the experiment condition, assuming the Model-1′s “two pairs of domain binding” is true. This c-Src Tyr-418 phosphorylation may weaken the total binding force between the α1 subunit and c-Src proposed in Model-1. However, it is unclear how Tyr-418 phosphorylation will affect the binding of c-Src KD to α1ND1 segment in this case. Furthermore, while using purified proteins as “bait” is a well-documented technique to study protein-protein interaction, the interaction of the α1 subunit and c-Src might also require other protein(s) and/or factors that are not present in cell-free systems.

What is more interesting is the comparison between SYF and SYF + c-Src cells (Fig. 6). The Src kinases-null SYF cells showed a different distribution pattern of the α1 subunit comparing with the SYF + c-Src cells. Specifically, in SYF cells, a large amount of “free” α1 subunit was present in BMH- and DTME-crosslinked samples, with or without DTT/SDS treatment (Fig. 6a,c). In contrast, in SYF + c-Src cells, there was only a small amount of “free” α1 subunit present in BMH- and DTME-crosslinked samples without DTT/SDS treatment, and there was an increase in “free” α1 subunit in DTME-crosslinked sample but not in BMH-crosslinked sample with DTT/SDS treatment. It is obvious that c-Src is a major interacting partner of the α1 subunit. The data also indicated that the α1 can be crosslinked with other protein(s) independent of c-Src, demonstrated by the α1 signal in other positions (Fig. 6a,c).

### The role of cav-1 in Na/K-ATPase signaling complex

A puzzlement of the present study is the interaction between the α1 subunit and cav-1. Cav-1 is a critical partner in Na/K-ATPase signaling^10,17,44^ in both Model-1 and Model-2. In BN-PAGE, both control and crosslinked samples showed very similar cav-1 immunoblotting bands (the 720-band) (Fig. 3). This high cav-1 molecular position is consistent with the reports that caveolin can form high molecular mass homo-oligomers by self-association^38,39^.

The discrepancy of the interaction between the α1 subunit and cav-1 might be generated by the detergent used in the present study. Digitonin and DDM (*n*-Dodecyl β-D-maltoside) are two mild detergents that are recommended and widely used in extraction of membrane protein complexes from the lipid bilayer without denaturation or dissociation of noncovalent protein-protein interactions^35,42,45^ for BN-PAGE analysis. While digitonin can better preserve native, fragile complexes, a slightly harsher DDM may induce complexes into sub-complexes^45–47^. There are two considerations to choose DDM over digitoxin. First, DDM is a better choice than other detergents in solubilization and preservation of membrane Na/K-ATPase activity^48,49^. Second, digitonin can inhibit Na/K-ATPase activity^50^ that would favor the Na/K-ATPase in E2(P) state. An E2(P)-prone conformation would release the c-Src KD from α1 ND1 segment in Model-1, leading to c-Src Tyr-418 phosphorylation. As a lipid-like nonionic detergent, DDM is efficient to solubilize cav-1^40,41^. It might also affect the binding of cav-1 with other proteins by replacing cav-1 from native lipids and/or introducing formation of sub-complexes^51–53^.

Since the α1 N- or C-terminal CBM only contains one Cys residue, which is not the key aromatic amino acid residue, and the Cys residues in cav-1 are outside of the caveolin scaffolding domain of cav-1^43^, a Cys-Cys interaction is not likely involved in cav-1 binding to α1 CBM. This is supported experimentally by the observations that non-crosslinked control sample showed similar cav-1 position comparing to crosslinked samples (Fig. 3a,b, Table 1), as well as that the interactions between the α1 and cav-1 (or between c-Src and cav-1) could be broken down by DTT/SDS treatment in BMH-crosslinked sample (Fig. 3c). Furthermore, in C2-9 cells, the α1 subunit and c-Src can form signaling complexes (Fig. 3b), as seen in LLC-PK1 cells (Fig. 3a).

Comparing the parent LLC-PK1 cells with cav-1-knockdown C2-9 cells (Fig. 7), DTT/SDS-treated BMH- and DTME-crosslinked samples showed that cav-1 affected the crosslinking of the α1 subunit with other proteins (Fig. 7a) but did not affect the crosslinking of c-Src with other proteins (Fig. 7b). It is known that caveolin, through its cytosolic caveolin scaffolding domain (aa 61–101), can directly bind to c-Src to negatively regulate c-Src auto-phosphorylation at Tyr-416^43^. Moreover, binding of caveolin to c-Src SH2 domain can phosphorylate caveolin at Tyr-14 which is outside caveolin scaffolding domain, and increase membrane association of activated c-Src^53,54^. In Model-2, Tyr-418 phosphorylation in purified c-Src^17^ suggested a dissociation of c-Src from caveolin. While auto-phosphorylation of c-Src could be regulated directly by c-Src-caveolin binding, our present data suggested that cav-1 does not directly interact with c-Src through a Cys-Cys mechanism under the experimental condition. Rather, binding of cav-1 to the α1 subunit anchors the Na/K-ATPase signaling complex within caveolae structure that is capable of concentrating signaling molecules such as c-Src^55^. Disruption of the caveolae structure by cav-1-knockdown moves the α1 subunit (and its signaling complex) out of the caveolae structure, thus disrupts/reduces α1/c-Src binding and leads to blocking of Na/K-ATPase signaling function^16^. This is also supported by the fact that Mβ-CD treatment increased Na/K-ATPase ion-transport activity in LLC-PK1 cells but not in C2-9 cells (Fig. 1). Considering that DDM might dissociate cav-1/caveolae from the α1/c-Src complex under present experimental settings, it could not exclude a direct interaction between cav-1 and the α1 subunit CBM, and/or between cav-1 and c-Src SH2 domain.

It was well-documented that other factors, like oxidative modification, are able to regulate the activity and signaling of the Na/K-ATPase, such as glutathionylation of Cys residue(s) of the α1 and β1 subunits^56,57^ as well as carbonylation modification of Pro224 residue of the α1 subunit^58,59^, which favored binding between the α1 subunit with c-Src. There is no doubt that the dynamic conformational change between E1(P) and E2(P) status, caused by any factor, would affect the binding status between the α1 subunit with c-Src. More interestingly, thiol modification also regulates the interaction between c-Src and α1 subunit. In the α1 subunit, substitution of Cys244 with alanine does not affect ouabain- and hypoxia-driven regulation of Src activity, but Сys 458 and 459 form the interaction interface between the α1 subunit and Src kinase and binding of glutathione to Сys 458 and 459 disrupts the interaction, suggesting that the Cys 458 and Cys459 is critical for the signaling activity of the Na/K-ATPase^60^. Since both crosslinkers (BMH and DTME) induce Cys-Cys crosslinking, it is highly possible that the Cys residues (Cys 244, 458, and 459) are also involved in the crosslinking reaction.

In summary, in live LLC-PK1 cells, the α1 subunit and c-Src form a protein-protein complex in native, non-crosslinked control condition. A Cys-Cys crosslinking approach demonstrated that there were interactions between the α1 subunit and c-Src, and between the α1 subunit and cav-1. While depletion of c-Src or cav-1 clearly reduced the involvement of the α1 subunit in the crosslinked protein complexes, depletion of cav-1 did not affect the interaction of c-Src with other proteins. Furthermore, capillary immunoblotting analysis showed multiple complexes with immuno-reactivity with the α1 subunit, c-Src, and cav-1, indicating the existence of multiple protein complexes containing different protein components. This may include, but not limited to, the reported interactions between the α1 subunit with other proteins such as adducin^61^, ankyrin^62^, FXYD proteins^63^, PI-3K^64^, 14–3–3 protein^65^, adaptor protein 1^66^, and Bcl-2^67^.

A disadvantage of using live cells and whole cell lysates is that it is not as clean as using overexpressed/purified recombinant proteins for protein-protein interaction studies, but with an obvious advantage of real cellular environment that reflects the real protein-protein interactions. Further studies in live cells with high resolution/separation method, under native condition, are needed to explore the components of different protein complexes and their functionalities.

## Materials and Methods

### Chemicals, antibodies, and gel electrophoresis supplies

All chemicals, except otherwise mentioned, were obtained from Sigma-Aldrich (St. Louis, MO). Monoclonal antibody against Na/K-ATPase α1 subunit (clone C464.6, Cat# 05-369) was from EMD Millipore Upstate (Billerica, MA). Monoclonal antibody against total c-Src (clone B-12, Cat# sc-8056) and HRP-conjugated secondary antibodies were from Santa Cruz (Santa Cruz, CA). Polyclonal antibodies against caveolin-1 (Cat# ab2910 for BN-PAGE immunoblotting and Cat#18199 for capillary immunoblotting) were from Abcam (Cambridge, MA).

Non-cleavable crosslinker BMH (bismaleimidohexane, Cat# 22330), cleavable crosslinker DTME (dithiobismaleimidoethane, Cat# 22335), and Halt Protease and Phosphatase Inhibitor Single-Use Cocktail (100X) were from ThermoFisher Scientific Pierce (Rockford, IL).

Novex 3–12% NativePAGE Bis-Tris gel, Novex Tris-Glycine gel (1.0 mm × 2D well), NativePAGE sample prep kit, NativeMark unstained protein standard, NativePAGE running buffer, NativePAGE cathode buffer additive, NuPAGE transfer buffer, and dithiothreitol (DTT) were from ThermoFisher Scientific Invitrogen (Calsbad, CA).

### Cell culture

Porcine renal proximal tubule LLC-PK1 cells (Cat# CL-101), triple Src kinase (c-Src, Yes, Fyn)-null mouse fibroblasts SYF cells (Cat# CRL-2459), and c-Src rescued SYF cells, SYF + c-Src cells (Cat# CRL-2498) were from ATCC (Manassas, VA). Cav-1-knockdown C2-9 cells were generated from LLC-PK1 cells^16^. The cells were cultured with DMEM (Dulbecco’s modified Eagle’s medium) with 10% fetal bovine serum (FBS), 100 U/ml penicillin, and 100 µg/ml streptomycin, in a 5% CO_2_-humidified incubator. Culture medium was changed daily until confluence. Cells were serum-starved for 16–18 h before treatment.

### ^86^Rb^+^ uptake assay

To evaluate the ion-transport activity of the Na/K‐ATPase, ouabain-sensitive ^86^Rb^+^ uptake assay was performed as previously described^65^. Briefly, to assess the effect of Mβ-CD, cells were pretreated with or without 10 mM Mβ-CD for 30 min at 37 °C. Prior to the initiation of ^86^Rb^+^ uptake, cellular Na^+^ was “clamped” with 20 μM monensin for 15 min to assure the measurement of maximal capacity of total active ^86^Rb^+^ uptake and to minimize the potential effect of changes in intracellular Na^+^. The assay was stopped 30 min after adding ^86^Rb^+^ (≈1 μCi/mL medium) by washing 3 times with ice‐cold 100 mM MgCl_2_ solution. In parallel, ouabain‐insensitive ^86^Rb^+^ uptake (cells were pretreated with 5 mM ouabain for 15 min) was measured in the presence of monensin. Ouabain‐sensitive ^86^Rb^+^ uptake was calculated by subtraction of ouabain‐insensitive ^86^Rb^+^ uptake from total ^86^Rb^+^ uptake, and then normalized by protein amount.

### Intracellular ATP level assay

ATP concentration was determined by CellTiter-Glo® Luminescent Cell Viability Assay Kit (Cat# G7570, Promega, Madison, WI) according to the manufacturer’s instruction. ATP concentrations were calculated by ATP concentration standard curve with ATP disodium salt (Cat# tlrl-atpl, InvivoGen, San Diego, CA) which was performed on the same plate. Cells were rinsed twice with culture medium to eliminate floating dead cells.

### Cell surface biotinylation assay

Cell surface biotinylation study was performed as we previously described^68^. EZ-Link Sulfo-NHS-SS-Biotin (Cat# 21331) and Streptavidin Agarose beads (Cat# 20353) were obtained from ThermoFisher Scientific.

### Protein carbonylation assay

Protein carbonylation study was performed as we previously described^58^.

### Protein crosslinking procedure in live cells

Crosslinking reaction was performed at room temperature, according to manufacturer’s instruction. Briefly, cells were rinsed 3x with 1X PBS buffer (with 1 mM EDTA). Freshly prepared BMH or DTME in DMSO (20 mM) were diluted to final concentration of 200 µM with 1X PBS buffer (with 1 mM EDTA). The cells were incubated with or without BMH or DTME for 60 min at room temperature in dark. The unreacted crosslinker were removed by rinsing the cells 3x with 1X PBS buffer. Cell pellets were put on ice and processed for whole cell lysate immediately.

### Whole cell lysate preparation for BN-PAGE

To prepare whole cell lysate under native condition for BN-PAGE, 1X NativePAGE sample buffer (with 2% DDM, n-dodecyl-β-D-maltoside; and Halt Protease and Phosphatase Inhibitor Cocktail) was added to cell pellets. After mixed by gently pipetting up and down for 8 times, the mixture was sit on ice for 30 min, and then centrifuged at 20,000 × g for 30 min at 4 °C. The supernatants were transferred/aliquotted to new tubes and stored in −80 °C until use. The sample protein concentration was determined by BCA protein assay kit (Bio-Rad).

### BN-PAGE analysis

Immediately prior to sample loading, the NativePAGE 5% G-250 sample additive was added to samples (at 1/4^th^ of detergent DDM concentration) and mixed. Electrophoresis was conducted according to manufacturer’s instruction, depending on following immunoblotting or gel staining.

### BN-PAGE/SDS-PAGE two-dimensional (2d) gel electrophoresis

To further separate protein components from Na/K-ATPase signaling complexes, a 2D (BN-PAGE as the first-dimension and SDS-PAGE as the second-dimension) gel electrophoresis approach was performed to identify proteins of interest (the Na/K-ATPase α1 subunit, c-Src, and cav-1). For each sample, two aliquots (with same amount of total proteins) were prepared separately. One aliquot was left aside on ice to keep protein complexes intact, and the other aliquot was mixed with DTT (final 100 mM) and SDS (final 1%, v/v) and incubated at 95 °C for 5 min to disrupt disulfide bond and negatively charge proteins. Briefly, the two sample aliquots (non-DTT/SDS-pretreated and DTT/SDS-pretreated) were first run on BN-PAGE (3–12% Bis-Tris gel) to separate protein complexes and proteins, and the whole lane slices were excised from the BN-PAGE gel. Before the second-dimensional SDS-PAGE, the whole lane slices were incubated for 10 min at room temperature in a SDS sample buffer (Tris 12.5 mM, SDS 4%, glycerol 20%, and bromophenol blue 0.02%)^37^, heated briefly in microwave (3X, 5 second each, depends on microwave power, avoid buffer boiling), and then incubated in the same SDS sample buffer for 15 min at room temperature. Each whole lane slice was loaded onto the 2D well of a Tris-Glycine gel, and electrophoresis was performed using standard SDS-PAGE protocol.

### Western blot with BN-PAGE and 2D-gel electrophoresis

For 1D BN-PAGE gels, proteins were transferred to Immobilon-P PVDF membrane (0.45 µm pore, EMD Millipore) with 1x NuPAGE transfer buffer, and then fixed proteins to PVDF membrane by incubating the PVDF membrane with 8% acetic acid (vol/vol) for 15 min at room temperature. For second-dimensional SDS-PAGE gels, proteins were transferred to PVDF membrane using standard protocol. For Western blot analysis, membranes were immunoblotted with indicated antibodies. Both primary and secondary antibodies were diluted with 5% non-fat milk in TBS-T (anti-α1 antibody, 1:2,000; anti-c-Src antibody, 1:500; anti-cav-1 antibody, 1:2,000; anti-mouse or rabbit secondary antibodies, 1:1000). Signal detection was performed with an enhanced chemiluminescence SuperSignal kit (Pierce) and FluorChem M detection system (ProteinSimple, San Jose, CA). Multiple exposures were analyzed to assure that the signals were within the range of the system. For BN-PAGE and BN-PAGE/SDS-PAGE 2D immunoblotting analysis (Figs. 3 and 4; please see original blotting images in Supplementary Information Figs. S1 and S2), the blot images with red background were converted to 256-gray color and negative mode, and the brightness and contrast of blots were slightly adjusted with FIJI Image J 1.51u. This aims to show clearer immunoblotting bands, since BN-PAGE immunoblotting usually show “smear” and/or weaker ECL signals with whole cell lysates prepared under native condition. For all images, any adjustment was applied equally across the entire image including controls.

### Mass spectrometer method

The gels were stained with Colloidal Coomassie dye G-250 based GelCode Blue stain reagent (ThermoFisher Scientific), according to the manufacture’s instruction. Briefly, the gels were fix in a fixing solution (50% ethanol, 40% double-distilled dd-H_2_O, and 10% acetic acid) for 1 h at room temperature, and then rehydrated with dd-H_2_O by 4 changes of dd-H_2_O, 30 min per change. The gels were then stained with GelCode Blue for at least 2 h, and de-stained in dd-H_2_O to enhance stain sensitivity until a desired clear background. The interested bands were processed for mass spectrometry analysis. After 1D BN-PAGE and GelCode Blue staining, two bands (the 720-band and 480-band), corresponding to the positive immune-reactivity for the α1 subunit, c-Src, and cav-1, were reduced, alkylated, and digested with trypsin. The extracted peptides were resolved on a nano column packed with 1.9um AQ C18 resin with 2-hour gradient run with Easy nLC 1200 UHPLC system (Thermo Scientific) and directly introduced into Orbitrap Fusion Lumos mass spectrometer (Thermo Scientific) using Flex nano spray ion source (Thermo Scientific). Flow rate was set at 300ul/min and column was heated at 45′C. All LC-MS/MS Tandem mass spectra were searched against porcine protein database (uniprot_sus_scrofa_pig_reviewed_04222019.fasta) assuming trypsin digestion by Byonic (Protein Metrics, San Carlos, CA; version ByonicNode in Proteome Discoverer 2.2.0.388) and Sequest (Thermo Fisher, San Jose, CA; version IseNode in Proteome Discoverer 2.2.0.388). Fragment ion mass tolerance and parent ion tolerance were set to 0.40 Da and 10.0 ppm (Monoisotopic), respectively. Scaffold (version Scaffold_4.8.9, Proteome Software Inc., Portland, OR) was used to validate MS/MS based peptide and protein identifications. Peptide identifications were positively established with more than 95.0% probability by the Scaffold Local FDR, Protein identifications were positively established with more than 99.0% probability and contained at least 2 identified peptides. Protein probabilities were assigned by the Protein Prophet algorithm^69^.

### Capillary-based immunoblotting analysis with wes system

After treatment with or without BMH or DTME, whole cell lysates were prepared with 1X NativePAGE sample buffer with Halt Protease and Phosphatase Inhibitor. After treatment with or without DTT/SDS, samples were separated in 66–440 kDa separation modules (Cat# SM-W008), immunoblotted with indicated primary antibodies, and then detected with anti-mouse or anti-rabbit detection modules (Cat# DM-001 and DM-002, using Wes system (ProteinSimple) according to manufacturer’s instruction. Primary antibodies were prepared with the antibody diluent provided in the detection module kit (anti-α1 antibody, 1:100; anti-c-Src antibody, 1:50; anti-cav-1 antibody, 1:50). The brightness and contrast of blots were slightly adjusted with Compass for SW software for Wes system (ProteinSimple). Please note that any adjustment does not change the spectrum of signals as shown in Figs. 2, 5, 6, and 7. One of the advantages of this system is that it can handle 24 samples (except protein ladder) in one 25-capillary cartridge for comparison with better separation range and resolution than traditional SDS-PAGE.

To compare the patterns of control, BMH- and DTME-crosslinked samples, two aliquots of each samples were treated without or with DTT (final concentration 40 mM) and SDS (final concentration 1%, v/v), at 60 °C for 30 min for the α1 subunit or 95 °C for 5 min for c-Src and cav-1.

### Statistical analysis

Data of ^86^Rb^+^ uptake assay, cell surface biotinylation, and protein carbonylation were tested for normality and then subjected to parametric analysis. GraphPad Prizm 8.0 (GraphPad Software, Inc; San Diego, CA) was used. Statistical significance was reported at the *P* < 0.05 and *P* < 0.01 levels. Values are given as MEAN ± SD.

## Supplementary information

Supplementary Information.

## Data Availability

The datasets generated during and/or analysed during the current study are available from the corresponding author on reasonable request.

## References

[1] Skou JC (1957). The influence of some cations on an adenosine triphosphatase from peripheral nerves. Biochim Biophys Acta.

[2] Aperia A (2007). New roles for an old enzyme: Na,K-ATPase emerges as an interesting drug target. J Intern Med.

[3] Bagrov AY, Shapiro JI, Fedorova OV (2009). Endogenous cardiotonic steroids: physiology, pharmacology, and novel therapeutic targets. Pharmacol Rev.

[4] Li Z, Xie Z (2009). The Na/K-ATPase/Src complex and cardiotonic steroid-activated protein kinase cascades. Pflugers Arch.

[5] Liang M, Cai T, Tian J, Qu W, Xie ZJ (2006). Functional Characterization of Src-interacting Na/K-ATPase Using RNA Interference Assay. J Biol Chem.

[6] Liu J, Xie ZJ (2010). The sodium pump and cardiotonic steroids-induced signal transduction protein kinases and calcium-signaling microdomain in regulation of transporter trafficking. Biochim Biophys Acta.

[7] Pierre SV, Xie Z (2006). The Na,K-ATPase receptor complex: its organization and membership. Cell Biochem Biophys.

[8] Tian J (2006). Binding of Src to Na+/K+-ATPase forms a functional signaling complex. Mol Biol Cell.

[9] Xie Z, Askari A (2002). Na(+)/K(+)-ATPase as a signal transducer. Eur J Biochem.

[10] Xie Z, Cai T (2003). Na+-K+–ATPase-mediated signal transduction: from protein interaction to cellular function. Mol Interv.

[11] Sweadner KJ, Donnet C (2001). Structural similarities of Na,K-ATPase and SERCA, the Ca(2+)-ATPase of the sarcoplasmic reticulum. Biochem J.

[12] Ferrandi M (2004). Organ hypertrophic signaling within caveolae membrane subdomains triggered by ouabain and antagonized by PST 2238. J Biol Chem.

[13] Ferrandi M, Molinari I, Bianchi G, Ferrari P (2006). Ouabain-dependent signaling in caveolae as a novel therapeutic target for hypertension. Cell Mol Biol (Noisy-le-grand).

[14] Kennedy DJ (2006). Central role for the cardiotonic steroid marinobufagenin in the pathogenesis of experimental uremic cardiomyopathy. Hypertension.

[15] Periyasamy SM (2005). Salt loading induces redistribution of the plasmalemmal Na/K-ATPase in proximal tubule cells. Kidney Int.

[16] Wang H (2004). Ouabain Assembles Signaling Cascades through the Caveolar Na+/K+-ATPase. Journal of Biological Chemistry.

[17] Yosef E, Katz A, Peleg Y, Mehlman T, Karlish SJD (2016). Do Src Kinase and Caveolin Interact Directly with Na,K-ATPase?. Journal of Biological Chemistry.

[18] Gable ME, Abdallah SL, Najjar SM, Liu L, Askari A (2014). Digitalis-induced cell signaling by the sodium pump: on the relation of Src to Na(+)/K(+)-ATPase. Biochem Biophys Res Commun.

[19] Weigand KM, Swarts HG, Fedosova NU, Russel FG, Koenderink JB (2012). Na,K-ATPase activity modulates Src activation: a role for ATP/ADP ratio. Biochim Biophys Acta.

[20] Bagrov AY, Shapiro JI (2008). Endogenous digitalis: pathophysiologic roles and therapeutic applications. Nat Clin Pract Nephrol.

[21] Mijatovic T, Dufrasne F, Kiss R (2012). Na+/K+-ATPase and cancer. Pharm Pat Anal.

[22] Schoner W (2002). Endogenous cardiac glycosides, a new class of steroid hormones. Eur J Biochem.

[23] Schoner W, Scheiner-Bobis G (2007). Endogenous and exogenous cardiac glycosides: their roles in hypertension, salt metabolism, and cell growth. Am J Physiol Cell Physiol.

[24] Wang HY, O’Doherty GA (2012). Modulators of Na/K-ATPase: a patent review. Expert Opin Ther Pat.

[25] Xie Z, Xie J (2005). The Na/K-ATPase-mediated signal transduction as a target for new drug development. Front Biosci.

[26] Yan Y, Shapiro JI (2016). The physiological and clinical importance of sodium potassium ATPase in cardiovascular diseases. Curr Opin Pharmacol.

[27] Yatime L (2009). P-type ATPases as drug targets: tools for medicine and science. Biochim Biophys Acta.

[28] Reinhard L, Tidow H, Clausen MJ, Nissen P (2013). Na(+),K (+)-ATPase as a docking station: protein-protein complexes of the Na(+),K (+)-ATPase. Cell Mol Life Sci.

[29] Liang M (2007). Identification of a Pool of Non-pumping Na/K-ATPase. Journal of Biological Chemistry.

[30] Liu J (2005). Ouabain-induced endocytosis of the plasmalemmal Na/K-ATPase in LLC-PK1 cells requires caveolin-1. Kidney Int.

[31] Klinghoffer RA, Sachsenmaier C, Cooper JA, Soriano P (1999). Src family kinases are required for integrin but not PDGFR signal transduction. EMBO J.

[32] Shimoni Y, Galili G (1996). Intramolecular Disulfide Bonds between Conserved Cysteines in Wheat Gliadins Control Their Deposition into Protein Bodies. Journal of Biological Chemistry.

[33] Shirai A (2008). Global analysis of gel mobility of proteins and its use in target identification. J Biol Chem.

[34] Rath A, Glibowicka M, Nadeau VG, Chen G, Deber CM (2009). Detergent binding explains anomalous SDS-PAGE migration of membrane proteins. Proc Natl Acad Sci USA.

[35] Schagger H, Cramer WA, von Jagow G (1994). Analysis of molecular masses and oligomeric states of protein complexes by blue native electrophoresis and isolation of membrane protein complexes by two-dimensional native electrophoresis. Anal Biochem.

[36] Schagger H, von Jagow G (1991). Blue native electrophoresis for isolation of membrane protein complexes in enzymatically active form. Anal Biochem.

[37] Swamy M, Siegers GM, Minguet S, Wollscheid B, Schamel WW (2006). Blue native polyacrylamide gel electrophoresis (BN-PAGE) for the identification and analysis of multiprotein complexes. Sci STKE.

[38] Monier S (1995). VIP21-caveolin, a membrane protein constituent of the caveolar coat, oligomerizes *in vivo* and *in vitro*. Mol Biol Cell.

[39] Sargiacomo M (1995). Oligomeric structure of caveolin: implications for caveolae membrane organization. Proc Natl Acad Sci USA.

[40] Han B, Tiwari A, Kenworthy AK (2015). Tagging strategies strongly affect the fate of overexpressed caveolin-1. Traffic.

[41] Fennessey CM, Sheng J, Rubin DH, McClain MS (2012). Oligomerization of Clostridium perfringens Epsilon Toxin Is Dependent upon Caveolins 1 and 2. PLOS ONE.

[42] Wittig I, Braun HP, Schagger H (2006). Blue native PAGE. Nat Protoc.

[43] Li S, Couet J, Lisanti MP (1996). Src Tyrosine Kinases, Gα Subunits, and H-Ras Share a Common Membrane-anchored Scaffolding Protein, Caveolin: CAVEOLIN BINDING NEGATIVELY REGULATES THE AUTO-ACTIVATION OF Src TYROSINE KINASES. Journal of Biological Chemistry.

[44] Wang Y (2014). Involvement of Na/K-ATPase in hydrogen peroxide-induced activation of the Src/ERK pathway in LLC-PK1 cells. Free Radic Biol Med.

[45] Reisinger V, Eichacker LA (2008). Solubilization of membrane protein complexes for blue native PAGE. J Proteomics.

[46] Brindley MA, Plemper RK (2010). Blue Native PAGE and Biomolecular Complementation Reveal a Tetrameric or Higher-Order Oligomer Organization of the Physiological Measles Virus Attachment Protein H. Journal of Virology.

[47] Reisinger V, Eichacker LA (2007). How to analyze protein complexes by 2D blue native SDS-PAGE. Proteomics.

[48] Cohen E (2005). Purification of Na+,K+-ATPase expressed in Pichia pastoris reveals an essential role of phospholipid-protein interactions. J Biol Chem.

[49] Habeck M, Kapri-Pardes E, Sharon M, Karlish SJD (2017). Specific phospholipid binding to Na,K-ATPase at two distinct sites. Proceedings of the National Academy of Sciences.

[50] Winter CG (1974). Digitonin as an uncoupler of the partial reactions of Na+, K+-ATPase. Ann N Y Acad Sci.

[51] Bueler SA, Rubinstein JL (2015). Vma9p need not be associated with the yeast V-ATPase for fully-coupled proton pumping activity *in vitro*. Biochemistry.

[52] Tokutsu R, Kato N, Bui KH, Ishikawa T, Minagawa J (2012). Revisiting the supramolecular organization of photosystem II in Chlamydomonas reinhardtii. J Biol Chem.

[53] Gottlieb-Abraham E (2013). Src-mediated caveolin-1 phosphorylation affects the targeting of active Src to specific membrane sites. Mol Biol Cell.

[54] Li S, Seitz R, Lisanti MP (1996). Phosphorylation of Caveolin by Src Tyrosine Kinases: THE α-ISOFORM OF CAVEOLIN IS SELECTIVELY PHOSPHORYLATED BY v-Src *IN VIVO*. Journal of Biological Chemistry.

[55] Schlegel A, Lisanti MP (2001). The caveolin triad: caveolae biogenesis, cholesterol trafficking, and signal transduction. Cytokine Growth Factor Rev.

[56] Figtree GA (2009). Reversible oxidative modification: a key mechanism of Na+-K+ pump regulation. Circ Res.

[57] Petrushanko IY (2012). S-glutathionylation of the Na,K-ATPase catalytic alpha subunit is a determinant of the enzyme redox sensitivity. J Biol Chem.

[58] Yan Y (2013). Involvement of reactive oxygen species in a feed-forward mechanism of Na/K-ATPase-mediated signaling transduction. J Biol Chem.

[59] Yan, Y. *et al*. Protein Carbonylation of an Amino Acid Residue of the Na/K-ATPase alpha1 Subunit Determines Na/K-ATPase Signaling and Sodium Transport in Renal Proximal Tubular Cells. *J Am Heart Assoc* **5**, 10.1161/JAHA.116.003675 (2016). 10.1161/JAHA.116.003675 PMC5079028 27613772

[60] Petrushanko IY (2017). Cysteine residues 244 and 458-459 within the catalytic subunit of Na,K-ATPase control the enzyme’s hydrolytic and signaling function under hypoxic conditions. Redox Biol.

[61] Ferrandi M (2010). Adducin- and ouabain-related gene variants predict the antihypertensive activity of rostafuroxin, part 1: experimental studies. Sci Transl Med.

[62] Nelson WJ, Veshnock PJ (1987). Ankyrin binding to (Na+ + K+)ATPase and implications for the organization of membrane domains in polarized cells. Nature.

[63] Geering K (2006). FXYD proteins: new regulators of Na-K-ATPase. Am J Physiol Renal Physiol.

[64] Yudowski GA (2000). Phosphoinositide-3 kinase binds to a proline-rich motif in the Na+, K+-ATPase alpha subunit and regulates its trafficking. Proc Natl Acad Sci USA.

[65] Efendiev R (2005). The 14-3-3 Protein Translates the NA+,K+-ATPase α1-Subunit Phosphorylation Signal into Binding and Activation of Phosphoinositide 3-Kinase during Endocytosis. Journal of Biological Chemistry.

[66] Efendiev R, Budu CE, Bertorello AM, Pedemonte CH (2008). G-protein-coupled receptor-mediated traffic of Na,K-ATPase to the plasma membrane requires the binding of adaptor protein 1 to a Tyr-255-based sequence in the alpha-subunit. J Biol Chem.

[67] Lauf PK, Alqahtani T, Flues K, Meller J, Adragna NC (2015). Interaction between Na-K-ATPase and Bcl-2 proteins BclXL and Bak. Am J Physiol Cell Physiol.

[68] Liu J (2002). Effects of cardiac glycosides on sodium pump expression and function in LLC-PK1 and MDCK cells. Kidney Int.

[69] Nesvizhskii AI, Keller A, Kolker E, Aebersold R (2003). A statistical model for identifying proteins by tandem mass spectrometry. Anal Chem.

