# Supplementary material for: The Na/K-ATPase α1 and c-Src form signaling complex under native condition: A crosslinking approach
Source: Sci Rep. 2020 Apr 7;10:6006. doi: 10.1038/s41598-020-61920-4 (PMC7138855; doi:10.1038/s41598-020-61920-4)
Supplement: Supplementary file 1 — Supplementary Information. [file 41598_2020_61920_MOESM1_ESM.pdf]

The Na/K-ATPase α1 and c-Src form signaling complex under native condition: A crosslinking approach

Ying Nie[1*], Fang Bai[1*], Muhammad A. Chaudhry[1], Rebecca Pratt[1], Joseph I. Shapiro[2], and Jiang Liu[1#]

Affiliations: From the [1] Dept. of Biomedical Sciences and [2] Dept. of Medicine, Joan C. Edwards School of Medicine, Marshall University, Huntington, WV 25755

* The authors contributed equally to this work (Y.N. and F.B.).

Correspondence to: Jiang Liu: Department of Biomedical Sciences, Joan C. Edwards School of Medicine, Marshall University, BBSC Room 241-S, 1700 3rd Ave., Huntington, WV 25755, USA. Tel. (304) 696-7359, Email liuj@marshall.edu.

Short Title: Binding of c-Src to Na/K-ATPase α1 in native state

Keywords: Na/K-ATPase, c-Src, caveolin, protein cross-linking, protein complex, signaling

---

Figure S1: BN-PAGE analysis of LLC-PK1 and C2-9 cells: *The α1 subunit and c-Src form protein-protein complex under native condition that is not dependent on cav-1.*

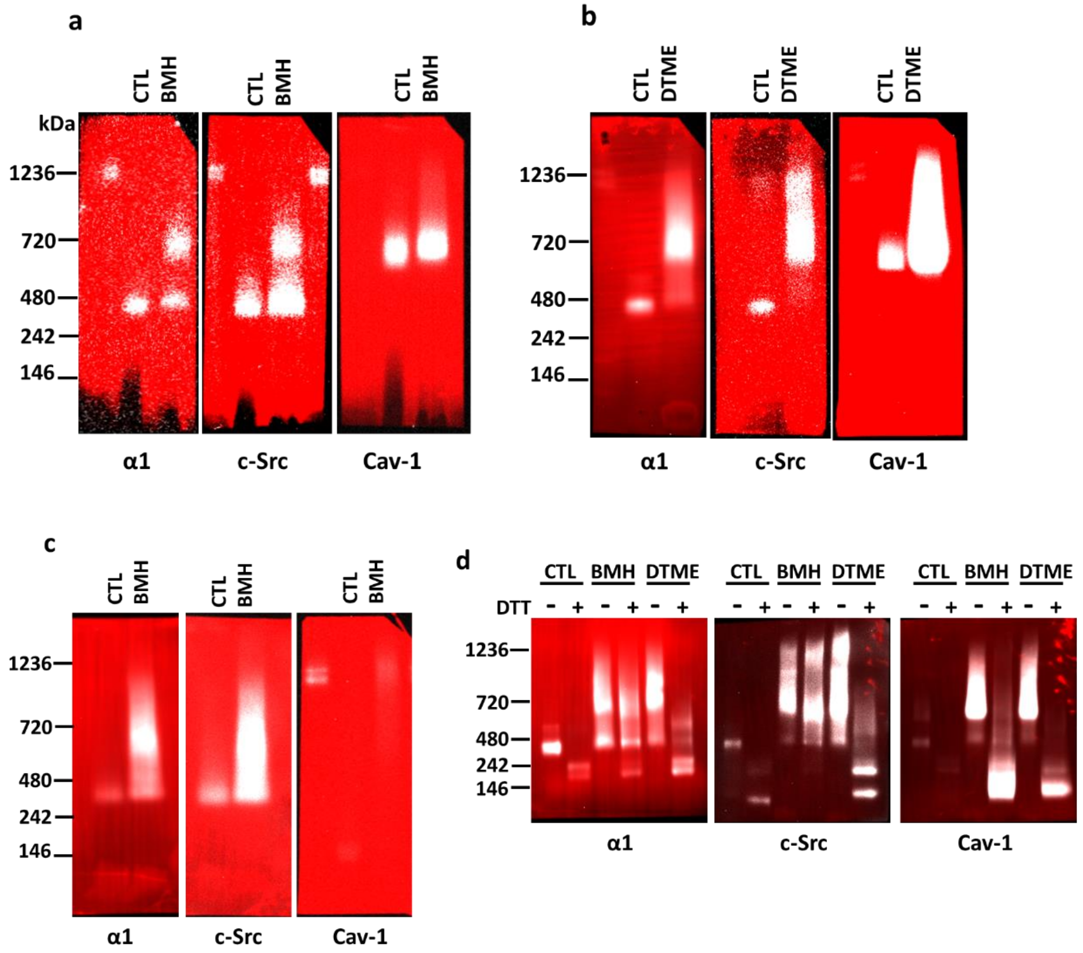

Crosslinking and preparation of whole cell lysate with Native-PAGE sample buffer were performed as described for BN-PAGE in the Materials and Methods. NativeMark unstained protein standard was located by Ponceau S staining after transferring to PVDF membrane. Control (CTL, with mocking crosslinking process without crosslinkers) and BMH- or DTME-crosslinked samples (25 μg protein/sample) were processed side-by-side and immunoblotted for the α1 subunit, c-Src, and cav-1, respectively. For **(a) (b) (c)**, the same CTL and crosslinked samples were separated into 3 groups (each group contains one CTL and crosslinked samples and separated by NativeMark protein standard

between groups) and run in the same gels. After transferring, the PVDF membrane was cut into the 3 groups and immunoblotted against each antibody individually. **(a)** BN-PAGE analysis of control (CTL) and BMH-crosslinked samples of LLC-PK1 cells**. (b)** BN-PAGE analysis of CTL and DTME-crosslinked samples of LLC-PK1 cells **(c)** BN-PAGE analysis of CTL and BMH-crosslinked samples of cav-1 depleted C2-9 cells. **(d)** Each sample (CTL, BMH- and DTME-crosslinked sample) of LLC-PK1 cells was treated with or without DTT/SDS (100 mM DTT with 1% SDS, final concentration, respectively). For DTT/SDS treatment, samples were heated at 60°C for 30 min (for the α1 subunit) or 95°C for 5 min (for c-Src and cav-1). FluorChem M detection system (ProteinSimple) was used to detect blot signals. FluorChem M imager system (ProteinSimple) was used to detect blot signals. n=3-4.

Figure S2: BN-PAGE/SDS-PAGE 2D gel electrophoresis analysis – the α1 subunit and c-Src form a protein complex:

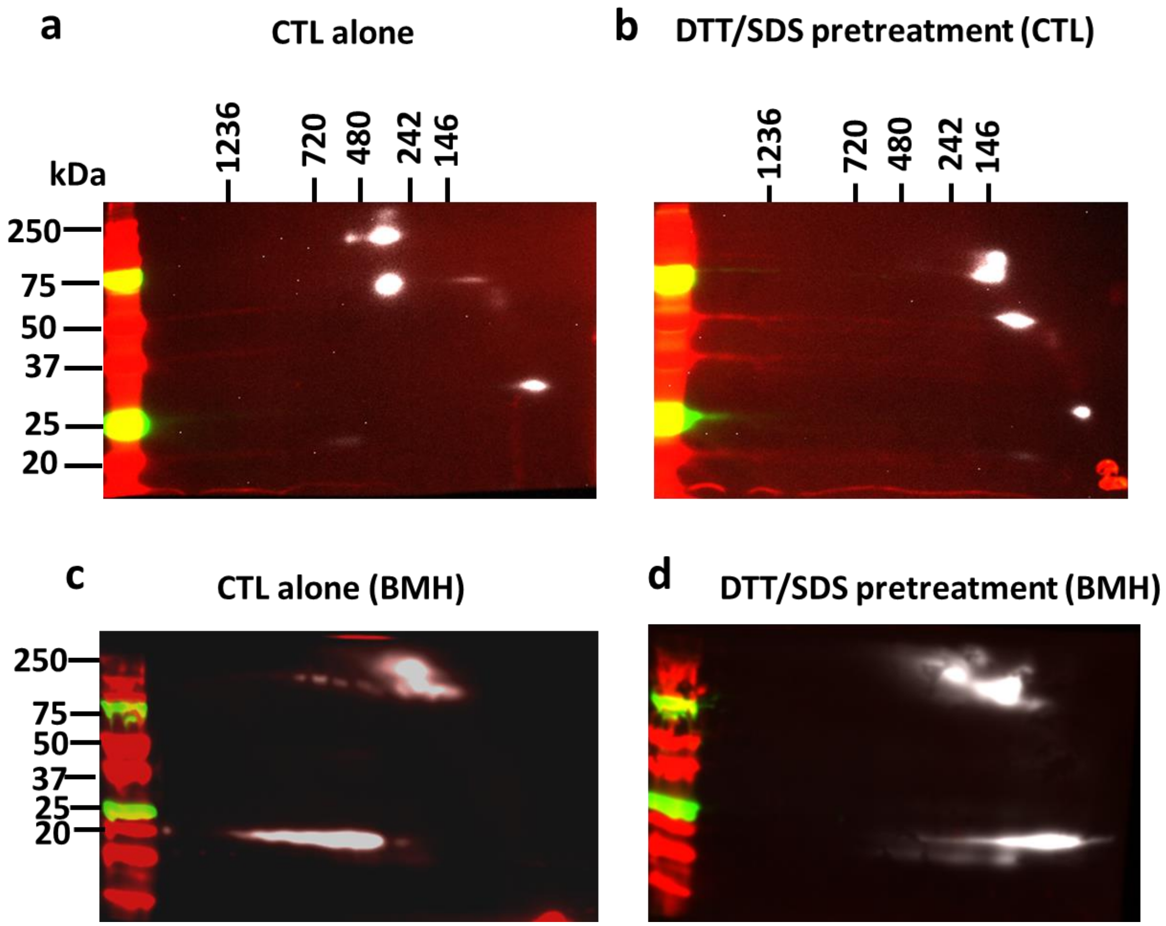

Preparation of whole cell lysate with Native-PAGE sample buffer were performed as described for BN-PAGE in the Materials and Methods. The experiments were performed as described for BN-PAGE/SDS-PAGE 2D Gel Electrophoresis in the Materials and Methods. Control and BMH-crosslinked samples of LLC-PK1 cells (50 µg/sample) was treated with or without DTT/SDS (100 mM DTT with 1% SDS, final concentration, respectively). For DTT/SDS treatment, samples were heated at 60°C for 30 min (for the α1 subunit) or 95°C for 5 min (for c-Src and cav-1). **(a)** Control sample without DTT/SDS pretreatment. **(b)** Control sample with DTT/SDS pretreatment. **(c)** BMH-crosslinked

sample without DTT/SDS pretreatment. **(d)** BMH-crosslinked sample with DTT/SDS pretreatment. Please note the vertical alignment of the α1 subunit and c-Src in **(a)** but not in **(b)**, which indicated that both proteins were from the same protein complex. Comparing to control **(a)** and **(b)**, disappearance of c-Src in **(c)** and **(d)** indicated the crosslinking between the α1 subunit and c-Src. n=3-4.
